# Brain derived neurotrophic factor and secreted amyloid precursor protein‐α response to moderate and high intensity exercise

**DOI:** 10.14814/phy2.70366

**Published:** 2025-05-22

**Authors:** Felicity S. E. Spencer, Richard J. Elsworthy, Leigh Breen, Jon R. B. Bishop, Samuel J. E. Lucas, Sarah Aldred

**Affiliations:** ^1^ School of Sport, Exercise and Rehabilitation Sciences, University of Birmingham Birmingham UK; ^2^ Birmingham Clinical Trials Unit Institute of Applied Health Research, Public Health Building, College of Medical and Dental Sciences, University of Birmingham Birmingham UK

**Keywords:** Alzheimer's disease, exercise, neurotrophic factors

## Abstract

Brain‐derived neurotrophic factor (BDNF) and secreted amyloid precursor protein‐alpha (sAPPα) promote neuronal growth but are lower in people with Alzheimer's Disease (AD). BDNF increases after exercise, but the response may vary with exercise intensity and across blood fractions. While sAPPα rises post‐exercise in animals, its response to aerobic exercise in humans is unclear. This study examined how BDNF and sAPPα respond to moderate and high‐intensity exercise in healthy young adult humans. Participants completed three exercise conditions: moderate (65% Watt maximum, 30 min), clinical‐HIIT (4 × 4 min, 90% Watt maximum), and all‐out‐HIIT (8 × 30 s, 150% Watt maximum). Blood‐based biomarkers were extracted from venous blood drawn from venepuncture at baseline, immediately after exercise, and after 30 min rest. BDNF concentration was measured in serum, plasma, platelet‐poor plasma, and platelets; sAPPα in serum via ELISAs. Data were primarily analyzed using mixed effects models. BDNF increased in plasma and platelet‐poor plasma across conditions but rose in serum only after clinical‐HIIT and moderate intensity exercise. BDNF concentration in platelets was unaffected. sAPPα increased after moderate and clinical‐HIIT sessions. Measurement of BDNF in serum alone may not fully represent the changes in BDNF post‐exercise. Further research is required in adults at risk of AD.

## 1 INTRODUCTION

Alzheimer's disease (AD) is a debilitating neurodegenerative condition that affects over 55 million people worldwide (Gustavsson et al., 2023). Regular exercise can reduce the risk of AD and delay disease progression (De la Rosa et al., 2020; Livingston et al., 2024). However, the use of varied exercise protocols in previous research studies means that the precise mechanism through which exercise can reduce AD risk remains unclear. As the societal burden of AD continues to grow alongside an aging population (Gustavsson et al., 2023), developing a better understanding of how AD risk can be reduced by lifestyle intervention is important.

Brain‐derived neurotrophic factor (BDNF) plays a key role in neuronal growth, maintenance, and repair; and as such, is vital for learning and memory (Bathina & Das, 2015; Erickson et al., 2012; Miranda et al., 2019). Lower levels of BDNF in people with AD have been identified in the hippocampus, and parietal, entorhinal, and frontal cortices, compared to healthy controls (Hock et al., 2000). This has been associated with AD pathology, including neuroinflammation, amyloid accumulation, and abnormal tau phosphorylation (Gao et al., 2022; Peng et al., 2005); and declining cognitive function (Ismail et al., 2020). Approximately 99% of BDNF in the periphery is stored in platelets (bound BDNF), with the remainder freely circulating in the blood (unbound BDNF) (Gejl et al., 2019). Peripheral and central BDNF concentrations are positively correlated (Klein et al., 2011), and there is some evidence that unbound BDNF can cross the blood–brain barrier (BBB) (Pan et al., 1998).

Individuals with neurodegenerative diseases, including AD, have significantly lower levels of BDNF in blood serum (measuring bound and unbound BDNF) than their healthy counterparts (Bathina & Das, 2015; Ng et al., 2019). The majority of research studies typically measure BDNF in only one blood fraction (Walsh & Tschakovsky, 2018); however, the concentration of BDNF in blood can be measured in several blood components. BDNF is most commonly measured in serum (total BDNF: bound and unbound), followed by plasma (unbound BDNF only) and platelets (bound BDNF only) (Dinoff et al., 2018). Although measurement of BDNF in plasma is thought to capture unbound BDNF only, platelets remain in this blood fraction, so sample processing may affect the amount of BDNF detected (Gejl et al., 2019). Therefore, the measurement of BDNF in platelet‐poor plasma in addition to plasma is recommended to ascertain a fuller understanding of BDNF response (Gejl et al., 2019).

As BDNF can be in the bound and unbound state, the fraction of blood chosen for analysis can affect the findings of a research study; for example, post exercise BDNF changes have been found in serum, but not plasma (Pareja‐Galeano et al., 2015), and it is largely unclear whether the concentration of BDNF in different blood fractions is related to each other. Previous research has found no correlation between BDNF levels in serum and plasma; however, there was a relationship between BDNF in plasma and platelet‐poor plasma (Gejl et al., 2019), but this was not measured after exercise.

Previous studies have also shown that circulating levels of BDNF are increased in healthy and clinical, young and old populations following acute bouts of aerobic or combined aerobic and strength exercise (e.g., (de Melo Coelho et al., 2013; Dinoff et al., 2018; Knaepen et al., 2010; Szuhany et al., 2015)). The mechanisms underpinning BDNF release following exercise are not completely clear. However, increases in blood lactate post‐exercise are associated with increased BDNF levels in serum and plasma (Müller et al., 2020), and infusion of lactate can stimulate BDNF release in serum (Schiffer et al., 2011), suggesting that there is a relationship between lactate and BDNF. However, lactate and BDNF levels after low‐intensity exercise are unrelated (Vega et al., 2006). Developing a better understanding of BDNF response post‐exercise in individuals with cognitive decline is important; however, from an ethical perspective, it is important to first study the effects of exercise on neurotrophic factors in a healthy population.

Changes in BDNF post‐exercise may be related to exercise intensity (Weaver et al., 2021), but findings vary by the blood fraction examined. For instance, continuous moderate intensity exercise and high intensity interval training (HIIT) have evoked similar post‐exercise increases in serum BDNF (Schmolesky et al., 2013). In contrast, higher intensity sprint intervals have been associated with a larger increase in plasma BDNF than lower intensity steady state exercise (Reycraft et al., 2020; Weaver et al., 2021). BDNF levels in platelets have been less well examined but have been associated with increases in BDNF in serum and plasma after exercise at V̇O_2_ max (Cho et al., 2012). BDNF responses to low‐intensity exercise across blood fractions have not been well characterized, but evidence suggests that BDNF levels in serum are unaffected by low‐intensity exercise (Hötting et al., 2016; Jeon & Ha, 2017; Piepmeier et al., 2020; Schmidt‐Kassow et al., 2012).

Alongside BDNF, previous research in animals has shown that other blood‐based biomarkers related to AD are altered by exercise bouts. Amyloid precursor protein (APP) is a transmembrane protein that can be cleaved in two distinct pathways with either neuroprotective or neurotoxic outcomes (Zhang et al., 2011). Cleavage of APP by the α‐secretase enzyme results in the production of secreted amyloid precursor protein‐α (sAPPα), which is neuroprotective and neurotrophic. sAPPα protects against excitotoxicity, and is important for neuronal plasticity (Zhang et al., 2011). In contrast, cleavage of APP by β‐secretase results in the production of amyloid‐β, which is toxic to neurones, and the build‐up of amyloid‐β plaques is a key hallmark of AD (Reiss et al., 2018). Therefore, higher levels of sAPPα are associated with shifting the cleavage of APP towards the neuroprotective pathway (MacPherson, 2017).

Findings from animal research suggest that exercise may increase sAPPα production. Treadmill running in mice is associated with increased α‐secretase production (Koo et al., 2017; Zhang et al., 2018). In concordance with this, acute bouts of exercise are associated with reduced levels of β‐secretase enzymes in the hippocampus in rodent models (Alkadhi & Dao, 2018; MacPherson et al., 2015).

Current studies investigating exercise‐based interventions in AD have heterogeneous exercise protocols (Frederiksen et al., 2017). There is a need to better understand how exercise in continuous vs. interval bouts may affect neurotrophic factors whilst considering the acceptability of the intervention. Typically, high intensity interval training (HIIT) is well‐tolerated in older adults and can produce a number of benefits (Marriott et al., 2021). For instance, the ‘clinical‐HIIT’ protocol (Rognmo et al., 2004) has shown to be acceptable, and produced improvements in older adults with cardiovascular disease (e.g., (Rognmo et al., 2012)). HIIT can be more time‐efficient than steady state exercise, and further investigation is warranted to develop targeted interventions.

It remains unclear how BDNF is affected by exercise intensity, and how exercise affects BDNF in different blood fractions. By developing a better understanding of these responses, there is scope to develop effective targeted exercise‐based interventions. Furthermore, despite promising findings in animals, it remains unclear how factors affecting amyloid processing respond to exercise in humans. Therefore, this study aims to investigate the effect of exercise intensity on BDNF and sAPPα, and to examine whether BDNF is differentially altered in serum, plasma, platelet‐poor plasma, and platelets. It was hypothesized that BDNF would increase pre to post exercise in all three conditions; though hypotheses were not specifically made about the individual blood fractions; and that sAPPα would increase post‐exercise.

## 2 METHOD

### 2.1 Ethical approval

The study conformed to the Declaration of Helsinki, except for registration in a database. This study was approved by the University of Birmingham Research Ethics Committee (ERN_17–1570). Prior to study participation, participants gave their informed consent in writing.

### 2.2 Participants

Participants were recruited via advertisement on e‐mailing lists at the University of Birmingham, student society Facebook pages, and flyers posted on university campus. This study was powered to detect a significant change in levels of BDNF in serum from pre‐ to post‐exercise (calculated by GPower v3.1). Data on mean (SD) difference in BDNF (pg/mL) from pre‐ to post‐exercise was taken from three related studies (Mang et al., 2014; Schmidt‐Kassow et al., 2012; Schmidt‐Kassow et al., 2014). The total number of participants required for 80% power, with a type I error rate of 5%, to detect a change (mean; SD) in BDNF levels ranged from 6 to 27. An average of these values gave a total number of participants of 17.33 (Knaepen et al., 2010). To account for an attrition rate of ~15% and missed samples, 3 additional participants were recruited, giving a total of 21 participants. The estimated standardized effect size ranged from 0.4 to 1.0, with the power to detect medium to large effects.

Participants were aged 18–45 years, with no history of cardiovascular, metabolic, neurological, or respiratory conditions, and were moderately physically active or physically active (as defined by the General Practice Physical Activity Questionnaire (GP‐PAQ) (2006); (Hwang et al., 2016)); equivalent to 3 h of exercise or more each week. Females were required to be taking any form of hormonal contraception to mitigate against hormonal fluctuations in the menstrual cycle that may affect BDNF levels (UK Government Department of Health and Social Care, 2013); as in (Pluchino et al., 2009). As this study was volunteer‐based and conducted at a University, the participant group was skewed towards younger and more active participants.

### 2.3 Procedure

This was a repeated‐measures study; except for the initial baseline session, session order was randomized. Before each session, participants were asked to avoid vigorous exercise and alcohol for at least 24 h. On the morning of the session, participants were instructed to avoid caffeine, arrive in a fasted state, and to ensure they were well hydrated with water only. All sessions took place in the morning, approximately 1 week apart and at approximately the same time for each participant. All sessions were conducted between 07:00 and 12:00 to limit the effects of circadian rhythm.

All sessions were completed on a cycle ergometer (Wattbike Atom X, Wattbike). Age, ethnicity (and duration of contraception: females only) were recorded by questionnaire. Body weight was measured via scales (kg) (Ohaus Champ II, Ohaus) and height by a measuring stick (cm) (Seca 213, Seca).

In the first session, participants were required to complete a V̇O_2_ max test. All participants were given time to familiarize themselves with the cycle ergometer before completing a 5‐min warm‐up at a self‐selected wattage. Adapted from (Schmidt‐Kassow et al., 2012), the test began at 70 W for females and 100 W for males, and increased by 30 W every 2 min until volitional exhaustion. Participants were allowed to listen to music and were verbally encouraged to cycle by the researcher(s) supervising the session. Rate of perceived exertion (RPE) was recorded every 2 min with Borg's RPE 6–20 scale (Mang et al., 2014). Heart rate (HR) was recorded every minute by telemetry (Polar H10, Polar).

Participants wore a facemask (Hans Rudolph 7450 Mask, Cranlea) to collect V̇O_2_ inhaled and V̇CO_2_ exhaled, to calculate V̇O_2_ max (Vyntus™ CPX metabolic cart, Vyaire medical). V̇O_2_ max was reached if two of the following were met: V̇O_2_ plateaued; RPE >17; volitional exhaustion; or attainment of age‐predicted maximal heart rate (Andersen, 1995). Watt max was determined as the power output achieved in the last partially (minimum 10 s into the stage) or fully complete stage of the V̇O_2_ max test.

**FIGURE 1:**
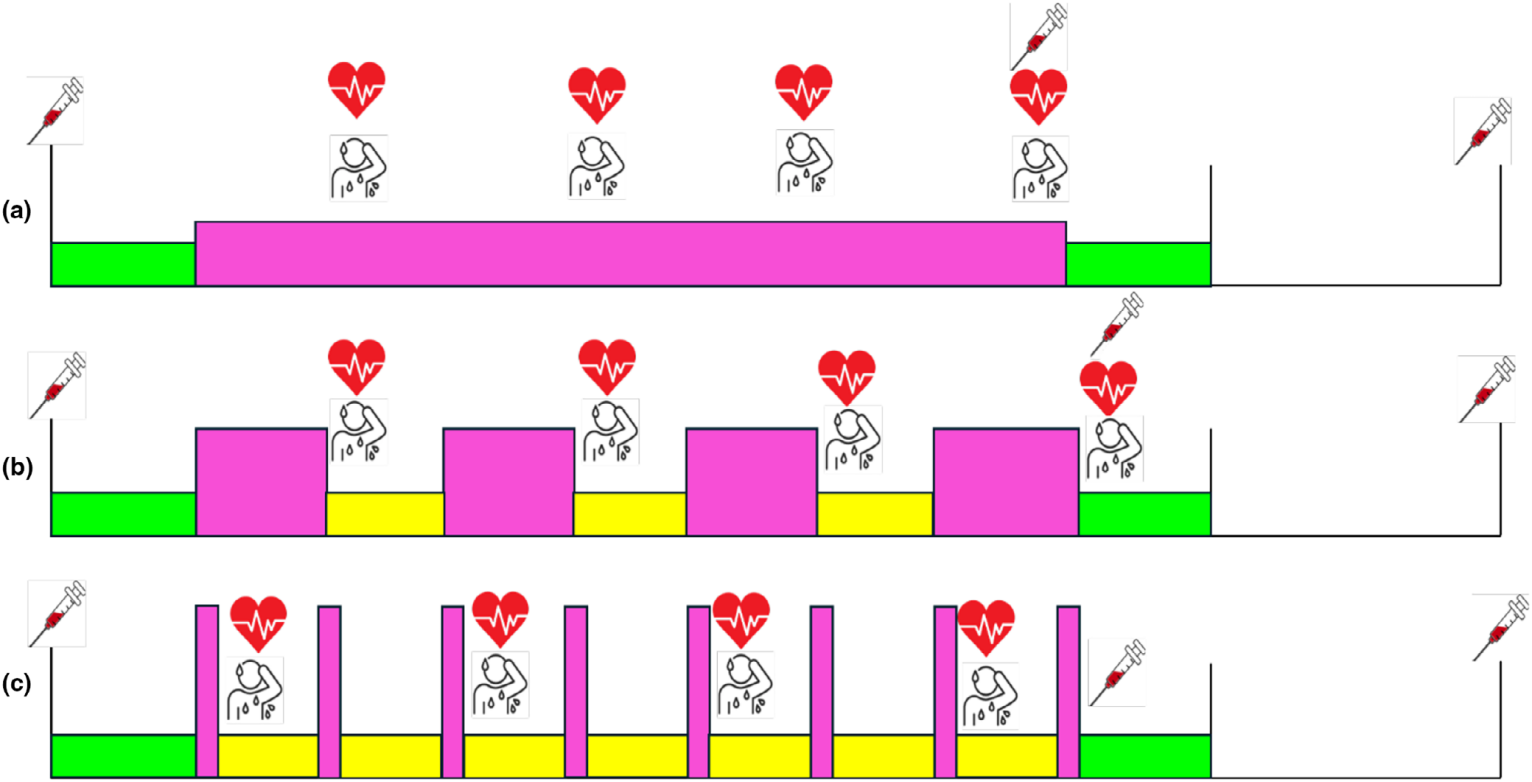
Schematic of the timings of measurements for each exercise session. (a) Moderate intensity. The green boxes represent warm‐up (5 min) and cool‐down (5 min), and the pink box represents 30 min of cycling at 65% of watt max. RPE and HR were measured every 7 min in the 30 min of moderate intensity exercise. Blood samples were taken prior to warm‐up (pre), immediately after moderate intensity exercise completion (post) and 30 min after exercise completion (delayed). (b) Clinical‐HIIT. The green boxes represent warm‐up (10 min) and cool‐down (5 min), and the pink boxes represent 4 × 4‐min bouts of cycling at 90% of watt max. The yellow boxes represent 4 × 3‐min bouts of active recovery. RPE and HR were measured immediately after completion of each 90% bout. Blood samples were taken prior to warm‐up (pre), immediately after completion of the last HIIT bout (post) and 30 min after exercise completion (delayed). (c) All‐out‐HIIT. The green boxes represent warm‐up (10 min) and cool‐down (5 min), and the pink boxes represent 8 × 30‐s bouts of cycling at 150% of watt max. The yellow boxes represent 8 × 3‐min 30‐s bouts of active recovery. RPE and HR were recorded immediately after completion of every other 150% bout, starting after bout 1. Blood samples were taken prior to warm‐up (pre), immediately after completion of the last HIIT bout (post) and 30 min after exercise completion (delayed).

The following three sessions (Figure 1) were completed by participants in a randomized order on a Wattbike (Wattbike Atom X, Wattbike) (generated by a random number generator controlled by a researcher independent to the study) and were standardized between participants using Watt max from session one.

Measures of RPE and heart rate were recorded at four regular intervals throughout each session (see Figure 1). The estimated work rate (metabolic/minutes; MET/min) was controlled between each session, calculated using the International Compendium of Physical Activities (Howley et al., 1995) as follows: moderate: 239 MET/min; clinical HIIT: 253.5 MET/min; all‐out HIIT: 206.5 MET/min. The total session duration was also controlled for as follows: moderate: 40 min; clinical‐HIIT: 42 min; and all‐out‐HIIT: 47 min. Total energy expenditure (kcals) for each session was calculated for each participant using the formula:

$$\begin{array}{c} \text{Total energy expenditure}\\ \;=\left(\left(\text{METs}\times 3.5\times \text{body weight}\;\left(\mathrm{kg}\right)\right)/200\right)\\ \;\times \text{minutes being active}. \end{array}$$

Average (SD) energy expenditure for each participant for each session was as follows: moderate: 319.95 (38.67) kcals; clinical‐HIIT: 314.99 (38.07) kcals; all‐out‐HIIT: 276.44 (33.41) kcals.

Blood samples were taken via venepuncture at baseline (pre; t1); immediately after completion of the exercise (moderate) or last HIIT bout (clinical‐HIIT and all‐out‐HIIT), prior to cool‐down (post; t2); and after 30 min of rest following completion of the exercise (delayed; t3) (see Figure 1). All samples were taken from the median cubital or median cephalic veins by a trained phlebotomist. Blood was collected into silica vacutainer (red top) (Vacutainer® BD) and EDTA vacutainer (purple top) (Vacutainer® BD) tubes at each time point (10 mL of blood in each tube at each time point; 60 mL blood per session). Immediately after collection, blood lactate (mmol/L) was ascertained from the sample (Lactate Pro 2, Arkray, BS48302).

Blood rested for 30 min at room temperature to allow for clotting, before both tubes were centrifuged at 800 ***g*** for 15 min at room temperature. The serum and plasma were then aliquoted. 2 mL (2 × 1 mL) of plasma from the EDTA vacutainer was centrifuged again at 3500 ***g*** for 15 min at room temperature for further separation into platelet‐poor plasma and platelets. After centrifugation, platelet‐poor plasma was aliquoted. 100 μL of a platelet lysis buffer with protease inhibitors (200 mM NaCl, 10 mM EDTA, 10 mM Na2HPO4, 0.5% NP40, 0.1% SDS and 1× protease inhibitors, Halt protease inhibitor cocktail, Thermo Scientific 78,425) was added to the platelets. All samples were stored at −80°C until analysis.

### 2.4 Sample analysis

BDNF levels in the four blood fractions (serum, plasma, platelet‐poor‐plasma, and platelets) at three timepoints (pre, post, and delayed) for each session (moderate, clinical‐HIIT, and all‐out‐HIIT) were determined by enzyme‐linked immunosorbent assay (ELISA) (Duoset ELISA, Bio‐techne DY999) as per manufacturer's instructions. Levels of sAPPα in serum were also determined by ELISA (IBL America, 27,734), though due to the exploratory nature of the analyses, a targeted approach was used, whereby only pre and post samples for the moderate and clinical‐HIIT sessions were analyzed. All samples were run in duplicate.

A Bicinchoninic acid (BCA) assay (Borg, 1982) (Thermo Scientific, 23,225) was conducted to determine the amount of platelet protein in each sample of platelets. The total amount of BDNF in each platelet sample was divided by the amount of platelet protein in each sample to produce the amount of BDNF per platelet, which was taken forward for statistical analysis.

### 2.5 Statistical analysis

To generate standard curves from the plate‐based assays, 4‐parameter logistic regression was used to plot known concentrations against optical absorbance at specified wavelengths, with www.myassays.com (RRID:SCR_016562).

Data were analyzed using R Studio v.4.2.2 (RRID:SCR_000432). All data are summarized as mean ± standard deviation unless otherwise specified. The level of alpha for statistical significance was *α* = 0.05.

BMI was calculated by dividing weight in kilograms (kg) by the square of height in meters (m^2^).

One‐way repeated measures analysis of variance (ANOVA) was used to examine differences in average RPE and HR (averaged across the 4 timepoints in each session) between the three conditions.

Multiple linear regressions were conducted to assess the relationship between BDNF immediately post exercise (in serum, plasma, platelet‐poor‐plasma, and per platelet) and average RPE; average HR; change in lactate; V̇O_2_ max; and sex across all three conditions. All predictors were entered into the models simultaneously, and BDNF levels at baseline were included in all models as covariates.

Linear mixed effects models were used (due to some missing data) to assess the effect of the three conditions on lactate levels and BDNF levels in serum, plasma, platelet‐poor plasma, and per platelet. Condition, time of sample, and the interaction between these variables were entered as fixed effects, and participant was entered as a random effect. Levels of blood lactate and BDNF were compared between all three conditions at all three timepoints (pre, post, delayed). Levels of sAPPα were compared between moderate and clinical‐HIIT conditions at pre and post timepoints. Models were compared to a null model to test model fit by analysis of variance (ANOVA), and model effect size was determined by conditional *R* ^2^. Post‐hoc Tukey's tests of multiple comparison were conducted to identify significant differences between conditions. Post‐hoc tests were performed examining comparisons within each condition (i.e., pre vs. post; post vs. delayed; pre vs. delayed only), and comparisons within each timepoint (i.e., clinical‐HIIT vs. all‐out‐HIIT at time 1; time 2; time 3 etc.). Cohen's d effect sizes were calculated for post‐hoc tests.

## 3 RESULTS

### 3.1 Demographics

There were 21 participants that entered the study (Table 1), with a mean age of 28.05 years (SD: 7.12). Participants were typically White (85.71% of total sample), with a healthy BMI (mean = 24.52, SD = 1.79), and above average aerobic fitness (mean V̇O_2_ max = 51.44 (SD = 8.59)) for their age and sex (approximate population averages in 20–29 year olds: male: 47.67 (SD = 6.49); female: 39.51 (SD = 8.76)) (mL/kg/min); (International Compendium of Physical Activities, 2011).

**TABLE 1:** Participant demographics for the total sample and disaggregated by sex.

	Total sample (*N* = 21)	Males (*n* = 17)	Females (*n* = 4)
Age (years)	28.05 (7.12)	28.47 (7.50)	26.25 (5.68)
Sex (% female)	19.1	0	100
Ethnicity (*N*, (%))			
White	18 (85.71)	14 (82.35)	4 (100)
Indian	2 (9.52)	2 (11.77)	0 (0)
Chinese	1 (4.76)	1 (5.89)	0 (0)
Weight (kg)	76.1 (9.2)	78.8 (7.7)	64.6 (4.7)
Height (cm)	176.0 (7.9)	178.5 (6.3)	165.2 (3.5)
BMI (kg/m^2^)	24.5 (1.8)	24.7 (1.8)	23.7 (1.5)
Contraceptive duration (months)	–	–	42.5 (35.2)
V̇O_2_ max (mL/kg/min); *n* = 20	51.4 (8.6)	53.0 (7.9)	45.3 (9.6)
Watt max (W)	294.3 (61.9)	311.9 (57.4)	220.0 (0.0)

*Note*: One participant's data for V̇O_2_ max was removed due to an equipment error. Statistics given are mean (SD) unless otherwise stated in the row header.

### 3.2 Heart rate and RPE

All participants met the criteria to determine V̇O_2_ max in the baseline session, where peak HR was 174 ± 21 beats/min and peak RPE score was 18 ± 1.

One participant dropped out of the study prior to the three testing sessions, citing an inability to adhere to the study protocol, leaving 20 participants who each attended all three follow‐up sessions.

RPE differed significantly between each session, *F* (2, 38) = 56.72, *p* < 0.001. Tukey's tests of multiple comparison demonstrated that each session was significantly different from the other: moderate/clinical‐HIIT, *t* (38) = 10.53, *p* < 0.001; moderate/all‐out‐HIIT, *t* (38) = 3.88, *p* = 0.00114; and clinical‐HIIT/all‐out‐HIIT, *t* (38) = −6.65, *p* < 0.001 (Table 2).

**TABLE 2:** Average peak and average RPE and HR over the three sessions.

	Moderate	Clinical‐HIIT	All‐out‐HIIT
Average Peak RPE	14.0 (1.5)	18.3 (1.4)	16.0 (1.8)
Average RPE	13.0 (1.6)	17.0 (1.8)	14.5 (2.3)
Average Peak HR	158 (12)	175 (10)	153 (15)
Average HR	153 (14)	165 (20)	140 (19)

*Note*: Values are mean (SD).

HR also varied significantly between each session, *F* (2, 38) = 18.77, *p* < 0.001. Post‐hoc Tukey's tests of multiple comparisons found that all three conditions differed significantly from each other: moderate/clinical‐HIIT, *t* (38) = 2.97, *p* = 0.0139; moderate/all‐out‐HIIT, *t* (38) = −3.16, *p* = 0.00859; clinical‐HIIT/all‐out‐HIIT, *t* (38) = −6.13, *p* < 0.001 (Table 2).

### 3.3 Lactate

**FIGURE 2:**
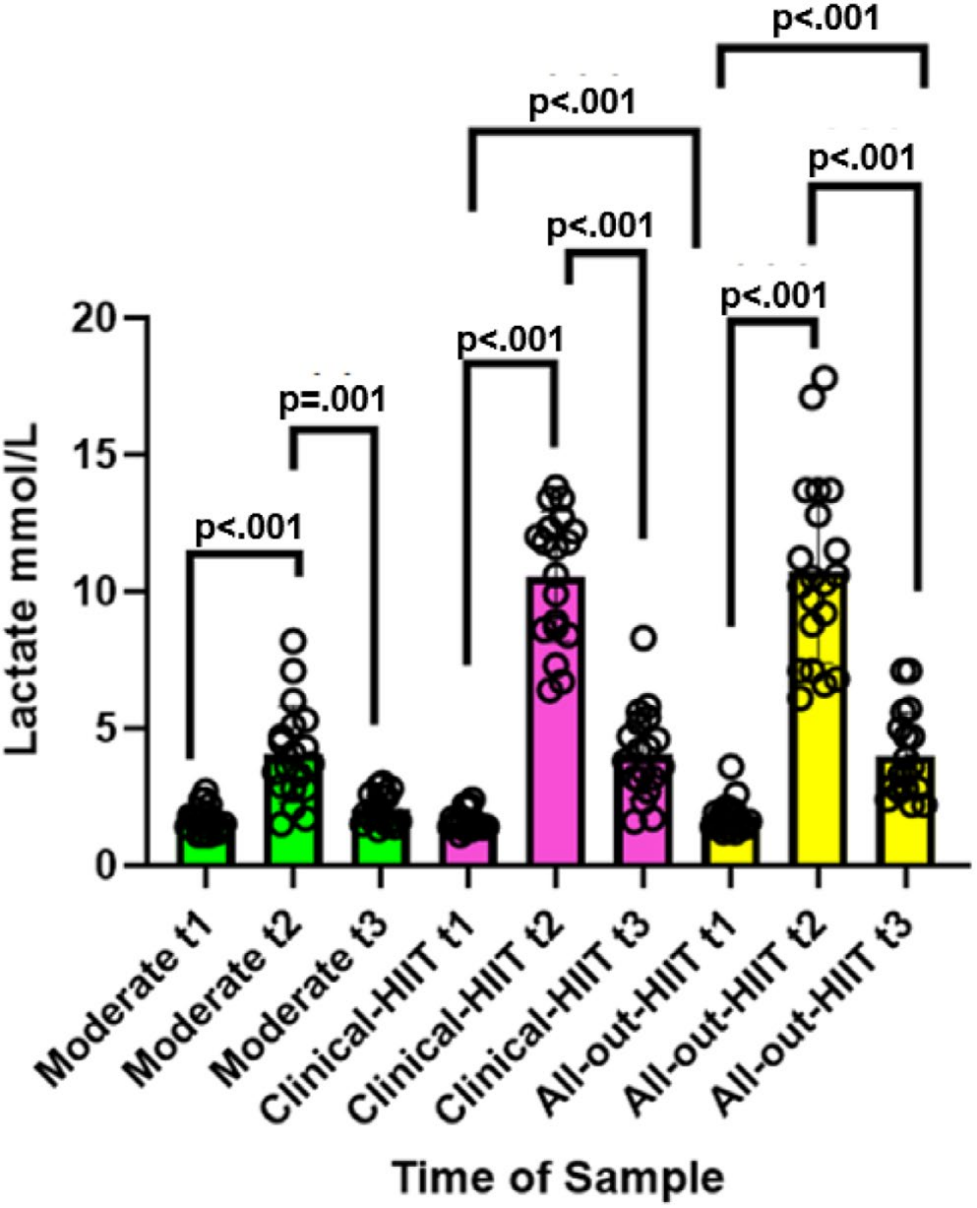
Amount of blood lactate (mmol/L). Lactate (mmol/L) for the moderate, clinical‐HIIT, and all‐out‐HIIT sessions. Note pre samples are denoted by t1; post by t2; and delayed by t3. Significant *p* values only shown, from Tukey's post‐hoc tests. Height of bars represents mean lactate (mmol/L) and error bars represent standard deviation. All *N* = 20 (*n* = 4 female), except for pre moderate (*N* = 19), post moderate (*N* = 18); delayed moderate (*N* = 18); pre clinical‐HIIT (*N* = 19), post clinical‐HIIT (*N* = 19), delayed clinical‐HIIT (*N* = 19) and delayed all‐out‐HIIT (*N* = 18) due to difficulties finding a suitable vein and equipment error.

Lactate levels were significantly different between each session, *χ* ^2^ (8) = 296.96, *p* < 0.001, conditional *R* ^2^ = 0.84 [95% CI] [0.80, 0.89]. (Figure 2; Table 3).

**TABLE 3:** Mean differences [95% CI] for blood lactate, and BDNF in serum, plasma, and platelet‐poor‐plasma, within sessions.

	Moderate	Clinical‐HIIT	All‐out‐HIIT
Lactate (mmol/L)			
Pre vs. Post	2.39 [1.56, 3.22]	8.96 [7.80, 10.12]	8.95 [7.31, 10.59]
Post vs. Delayed	−2.00 [−2.70, −1.30]	−6.52 [−7.41, −5.62]	−6.26 [−7.34, −5.17]
Pre vs. Delayed	0.34 [0.03, 0.65]	2.44 [1.60, 3.28]	2.19 [1.28, 3.10]
Serum BDNF (pg/mL)			
Pre vs. Post	4730 [1030, 8430]	5580 [2520, 8640]	3580 [498, 6660]
Post vs. Delayed	−4290 [−8740, 163]	−3460 [−6790, −140]	−3070 [−5880, −264]
Pre vs. Delayed	333 [−3150, 3820]	1760 [−2670, 6190]	−337.61 [−3660, 2980]
Plasma BDNF (pg/mL)			
Pre vs. Post	1610 [1100, 2120]	1730 [767, 2690]	1310 [586, 2040]
Post vs. Delayed	−2090 [−2820, −1370]	−2040 [−2900, −1190]	−1040 [−1650, −422]
Pre vs. Delayed	−517 [−1300, 272]	−306 [−1340, 728]	288 [−568, 1150]
Platelet‐Poor‐Plasma BDNF (pg/mL)			
Pre vs. Post	1460 [697, 2220]	1690 [608, 2770]	1510 [628, 2390]
Post vs. Delayed	−1060 [−2040, −74.0]	−2062 [−2760, −1360]	−727 [−1820, 361]
Pre vs. Delayed	359 [−189, 907]	−335 [−1090, 418]	845 [−1.64, 1690]
BDNF Per Platelet (pg/mL)			
Pre vs. Post	−3.46 [−1990, 1980]	−1020 [−2770, 732]	−456 [−1710, 800]
Post vs. Delayed	347 [−536, 1230]	−180 [−1660, 1300]	2770 [−4610, 1010]
Pre vs. Delayed	−348 [−2260, 1570]	−660 [−2040, 720]	−2120 [−4530, 8770]

*Note*: All values reported to 3 significant figures.

After applying Tukey's tests of multiple comparisons, lactate differed significantly between all within‐session comparisons except pre versus delayed in the moderate session (all, *p* < 0.05, Table S1). All effect sizes were large, all *d* > 0.8; except for the pre versus delayed comparison in the moderate session, *d* = −0.27 [−0.92, 0.38] (see Table S1). Compared to the moderate condition, Lactate levels were significantly higher at both the immediately post‐exercise and delayed time points in both the all‐out‐HIIT and the clinical‐HIIT conditions (all *p* < 0.05, Table 4).

**TABLE 4:** Results of Tukey's tests of multiple comparisons for blood lactate, and BDNF in serum, plasma, platelet‐poor‐plasma, and sAPPα in serum between conditions.

	Moderate	Clinical‐HIIT	All‐out‐HIIT	Clinical‐HIIT vs. moderate	All‐out‐HIIT vs. moderate	All‐out‐HIIT vs. clinical‐HIIT
Lactate (mmol/L)						
Pre	1.67 (0.47)	1.59 (0.36)	1.77 (0.56)	Mean Difference = 0.03 [−1.24, 1.18], *p* = 0.998, *d* = −0.02 [−0.66, 0.62]	Mean Difference = 0.15 [−1.04, 1.34], *p* = 0.951, *d* = 0.10 [−0.54, 0.73]	Mean Difference = 0.18 [−1.01, 1.37], *p* = 0.931, *d* = 0.12 [−0.52, 0.75]
Post	4.06 (1.75)	10.55 (2.37)	10.72 (3.33)	**Mean Difference = 6.54 5.33,7.74], *p* < 0.001, *d* = 4.18 [3.90, 4.97]**	**Mean Difference = 6.71 [5.52, 7.90], *p* < 0.001, *d* = 4.29 [3.50, 5.08]**	Mean Difference = −0.17 [−1.02, 1.36], *p* = 0.937, *d* = 0.11 [−0.52, 0.75]
Delayed	2.03 (0.55)	4.04 (1.61)	4.00 (1.58)	**Mean Difference = 1.99 [0.77, 3.21], *p* = 0.000512, *d* = 1.27 [0.60, 1.94]**	**Mean Difference = 2.02 [0.78, 3.25], *p* = 0.000503, *d* = 1.29 [0.61, 1.97]**	Mean Difference = 0.03 [−1.12, 1.25], *p* = 0.999, *d* = 0.02 [−0.64, 0.67]
BDNF in Serum (pg/mL)						
Pre	16371.20 (5132.59)	20259.20 (8168.20)	16712.85 (5561.86)	Mean Difference = 3888.00 [−100.14, 7876.14] *p* = 0.0578, *d* = 0.73 [0.10, 1.37]	Mean Difference = 341.65 [−3646.49, 4329.79], *p* = 0.978, *d* = 0.06 [−0.57, 0.69]	Mean Difference = −3546.35 [−7534.49, 441.79], *p* = 0.0922, *d* = −0.67 [−1.30, −0.03]
Post	20933.22 (8910.91)	25838.50 (8016.05)	20292.50 (5477.86)	**Mean Difference = 4746.96 [635.64, 8858.27], *p* = 0.0192, *d* = 0.89 [0.23, 1.55]**	Mean Difference = −799.04 [−4910.36, 3312.27], *p* = 0.890, *d* = −0.15 [−0.80, 0.50]	**Mean Difference = −5546.00 [−9534.14, −1557.86], *p* = 0.00355, *d* = −1.04 [−1.68, −0.40]**
Delayed	17257.00 (5270.99)	22699.53 (8179.51)	16787.22 (4292.87)	**Mean Difference = 5365.47 [1063.30, 9667.63], *p* = 0.0102, *d* = 1.01 [0.32, 1.70]**	Mean Difference = −12.03 [−4360.57, 4336.52], *p* = 0.999, *d* = −0.00 [−0.69, 0.68]	**Mean Difference = −5377.50 [−9547.10, −1207.89], *p* = 0.00754, *d* = −1.01 [−1.68, −0.34]**
BDNF in Plasma (pg/mL)						
Pre	6721.65 (2598.52)	7158.15 (3020.53)	7206.40 (3363.18)	Mean Difference = 436.50 [−828.99, 1701.99], *p* = 0.693, *d* = 0.26 [−0.38, 0.90]	Mean Difference = 484.75 [−780.75, 1750.24], *p* = 0.637, *d* = 0.19 [−0.35, 0.93]	Mean Difference = 48.25 [−1217.24, 1313.74], *p* = 0.996, *d* = 0.029 [−0.61, 0.67]
Post	8469.89 (2594.36)	8884.40 (2185.04)	8520.15 (2888.66)	Mean Difference = 417.37 [−887.74, 1722.47], *p* = 0.730, *d* = 0.25 [−0.41, 0.91]	Mean Difference = 53.12 [−1251.99, 1358.22], *p* = 0.995, *d* = 0.03 [−0.63, 0.69]	Mean Difference = −364.25 [−1629.74, 901.24], *p* = 0.775, *d* = −0.22 [−0.86, 0.42]
Delayed	6329.25 (2846.78)	6898.95 (2727.89)	7115.44 (3045.34)	Mean Difference = 487.73 [−882.95, 1858.41], *p* = 0.677, *d* = 0.29 [−0.40, 0.98]	Mean Difference = 1001.21 [−378.88, 2381.30], *p* = 0.202, *d* = 0.59 [−0.11, 1.29]	Mean Difference = 513.48 [−810.14, 1837.09], *p* = 0.629, *d* = 0.30 [−0.37, 0.97]
BDNF in Platelet‐Poor‐Plasma (pg/mL)						
Pre	2064.54 (1949.04)	2468.01 (2284.49)	2137.36 (1738.46)	Mean Difference = 464.52 [−600.91, 1529.94], *p* = 0.558, *d* = 0.33 [−0.32, 0.98]	Mean Difference = 133.87 [−931.56, 1199.29], *p* = 0.952, *d* = 0.10 [−0.55, 0.74]	Mean Difference = −330.65 [−1380.29, 718.99], *p* = 0.736, *d* = −0.24 [−0.87, 0.40]
Post	3209.23 (2271.39)	4156.82 (2127.88)	3648.97 (2700.13)	Mean Difference = 842.97 [−239.43, 1925.37], *p* = 0.159, *d* = 0.60 [−0.06, 1.26]	Mean Difference = 335.12 [−747.28, 1417.52], *p* = 0.744, *d* = 0.24 [−0.42, 0.90]	Mean Difference = −507.85 [−1557.49, 541.79], *p* = 0.487, *d* = 0.36 [−0.10, 0.27]
Delayed	2310.82 (2083.91)	2228.69 (1640.20)	2905.54 (2114.68)	Mean Difference = −159.82 [−1315.04, 995.39], *p* = 0.943, *d* = −0.11 [−0.81, 0.59]	Mean Difference = 691.88 [−475.41, 1859.18], *p* = 0.342, *d* = 0.49 [−0.21, 1.20]	Mean Difference = 851.71 [−246.54, 1949.95], *p* = 0.161, *d* = 0.61 [−0.06, 1.28]

	Moderate	Clinical‐HIIT	Clinical‐HIIT vs. Moderate
sAPPα in Serum (ng/mL)			
Pre	13.82 (11.37)	11.97 (6.10)	−1.86 [−9.00, 5.29], *p* = 0.605, *d* = −0.17 [−0.80, 0.47]
Post	23.37 (17.35)	31.85 (16.59)	**7.58 [0.08, 15.07], *p* = 0.0477, *d* = 0.67 [−0.00, 1.35]**

*Note*: The last three columns (last column only for sAPPα in Serum) are all model‐based marginal estimates of mean differences and not simply differences in observed means. *p* values <0.05 are formatted in bold.

### 3.4 BDNF

There was no evidence of relationships between average RPE, average HR, change in lactate, V̇O_2_ max, or sex and BDNF post exercise in serum, plasma, platelet‐poor plasma, or per platelet (Table 5).

**TABLE 5:** Results of linear regression models (*β* [95% CI]) examining the relationship between BDNF post‐exercise, and average RPE, average HR, change in lactate, V̇O_2_ max, and sex, across serum, plasma, platelet‐poor‐plasma, and per platelet.

	Blood fraction			
	Serum	Plasma	Platelet‐poor‐plasma	Per platelet
Variable				
Pre BDNF	0.58 [0.28, 0.89, *p* = 0.000353	0.70 [0.56, 0.83], *p* < 0.001	0.73 [0.42, 1.03], *p* < 0.001	0.55 [0.25, 0.85], *p* = 0.000671
Average RPE	605.66 [−536.83, 1748.14], *p = 0*.291	−36.28 [−270.19, 197.64], *p = 0*.756	−58.18 [−396.15, 279.80], *p = 0*.730	−301.13 [−809.20, 206.95], *p = 0*.237
Average HR	43.81 [−87.72, 175.34], *p = 0*.506	2.99 [−24.17, 30.15], *p = 0*.826	5.59 [−33.79, 44.96], *p = 0.776*	−13.08 [−69.78, 43.62], *p = 0*.643
Pre Lactate	−1758.43 [−6009.85, 2492.98], *p* = 0.409	−519.84 [−1391.08, 351.41], *p* = 0.236	−214.22 [−1483.41, 1054.97], *p* = 0.737	−67.99 [−1885.58, 1749.60], *p* = 0.940
Post Lactate	−190.58 [−795.25, 414.09], *p* = 0.529	63.22 [−63.13, 189.57], *p* = 0.319	66.68 [−116.09, 249.44], *p* = 0.466	12.12 [−267.50, 291.75], *p* = 0.931
V̇O_2_ max	49.15 [−183.25, 281.56], *p* = 0.672	−10.79 [−57.16, 35.58], *p* = 0.642	33.28 [−34.82, 101.39], *p* = 0.330	−41.56 [−141.00, 57.89], *p* = 0.403
Sex	6.59 [−4574.54, 4587.73], *p* = 0.997	−603.89 [−1549.42, 341.64], *p* = 0.205	−61.07 [−1444.96, 1322.81], *p* = 0.929	1074.24 [−1039.00, 3187.49], *p* = 0.310
Model Fit Summary				
	*R* ^2^ = 0.33, *F* (7, 45) = 3.31, *p* = 0.00627	*R* ^2^ = 0.75, *F* (7, 45) = 3.31, *p* < 0.001	*R* ^2^ = 0.38, *F* (7, 44) = 3.87, *p* = 0.00231	*R* ^2^ = 0.28, *F* (7, 39) = 2.16, *p* = 0.0599

*Note*: Each Pre BDNF refers to the levels of baseline BDNF in each blood fraction.

**FIGURE 3:**
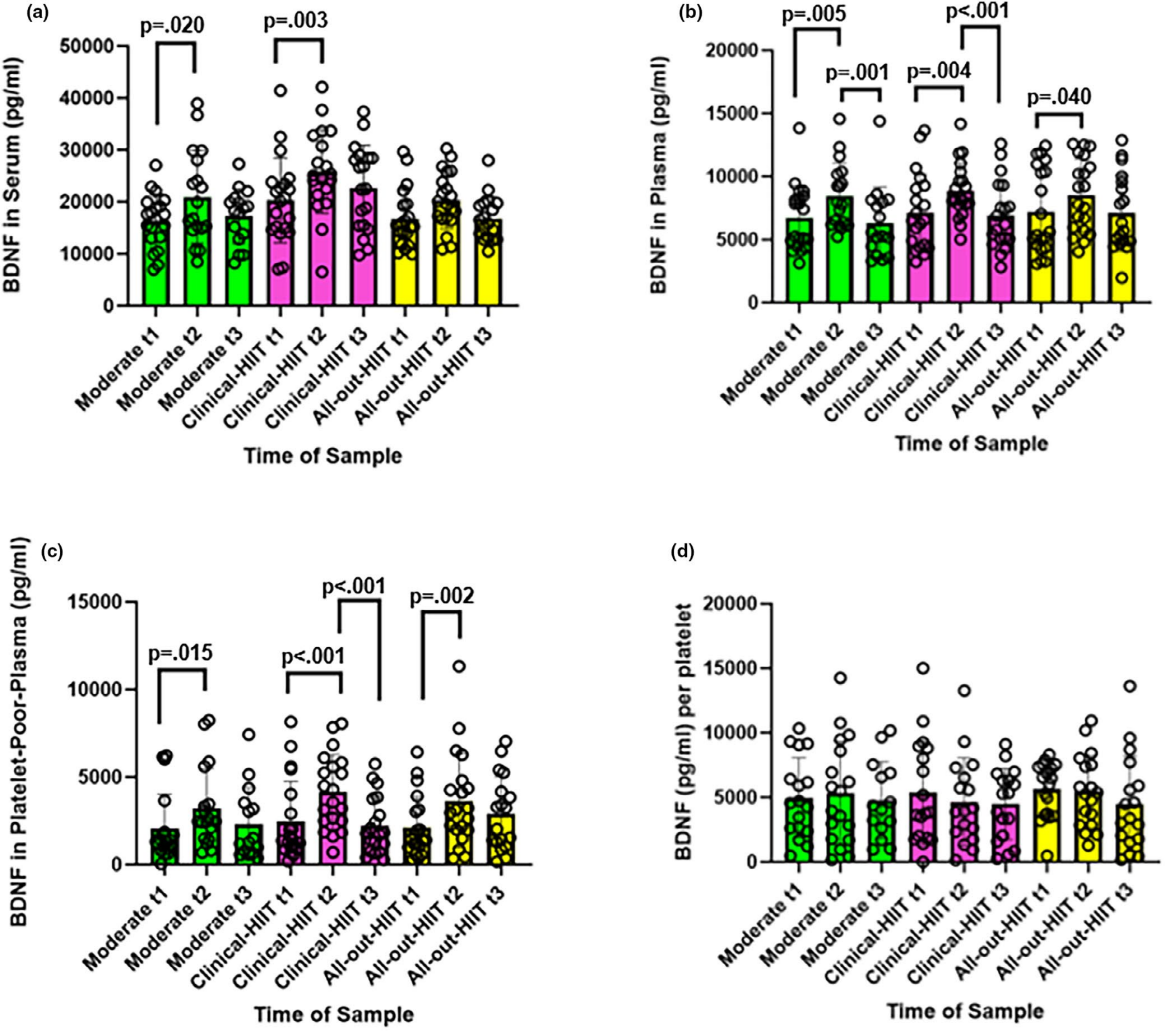
Amount of BDNF across blood fractions (pg/mL). BDNF (pg/mL) for the moderate, clinical‐HIIT, and all‐out‐HIIT sessions. Note pre samples are denoted by t1; post by t2; and delayed by t3. Significant *p* values are only shown from Tukey's post‐hoc tests. The height of bars represents mean BDNF (pg/mL) and error bars represent standard deviation. Panels display the amount of BDNF. (a) in serum (pg/mL); all *N* = 20 (*n* = 4 female), except for moderate t2 (*N* = 18; *n* = 3 female); moderate t3 (*N* = 16; *n* = 3 female); clinical‐HIIT t3 (*N* = 19), and all‐out‐HIIT t3 (*N* = 18), due to difficulties finding a suitable vein. (b) in plasma (pg/mL); all *N* = 20, except for moderate t2 (*N* = 18; *n* = 13 female); moderate t3 (*N* = 16; *n* = 3 female); clinical‐HIIT t3 (*N* = 19), and all‐out‐HIIT t3 (*N* = 18), due to difficulties finding a suitable vein. (c) in platelet‐poor plasma (pg/mL); all *N* = 20 (*n* = 4 female), except for moderate t1 (*N* = 19); moderate t2 (*N* = 18; *n* = 3 female); moderate t3 (*N* = 15; *n* = 3 female); clinical‐HIIT t3 (*N* = 19); and all‐out‐HIIT t3 (*N* = 18), due to difficulties finding a suitable vein and two samples containing levels of protein below detectable levels. (d) per platelet (pg/mL); all *n* = 4 female except where specified; moderate t1 (*N* = 17); moderate t2 (*N* = 18, *n* = 3 female); moderate t3 (*N* = 14, *n* = 3 female); clinical‐HIIT t1 (*N* = 18); clinical‐HIIT t2 (*N* = 17, *n* = 3 female); clinical‐HIIT t3 (*N* = 17, *n* = 3 female); all‐out‐HIIT t1 (*N* = 19); all‐out‐HIIT t2 (*N* = 18); and all‐out‐HIIT t3 (*N* = 18); due to difficulties finding a suitable vein, and 14 samples containing levels of protein below detectable levels.

The mixed effect model demonstrated that levels of BDNF in serum were significantly different between the three conditions, *χ* ^2^ (8) = 49.81, *p* < 0.001, conditional *R* ^2^ = 0.49 [0.25, 0.63]. Levels of BDNF in serum between pre and post samples for the clinical‐HIIT and moderate sessions increased by 4730 [1030, 8430] and 5580 [2520, 8640] pg/mL respectively (Table 3). Tukey's post‐hoc tests found that these differences were significant, *t*(143) = −2.72, *p* = 0.0200; *d* = 0.89 [−1.54, −0.23], and *t*(143) = −3.31, *p* = 0.00333; *d* = −1.05 [−1.69, −0.41], respectively. No other tests within conditions showed significant differences (Table 3; Table S1; Figure 3a). Levels of BDNF were significantly higher in the clinical‐HIIT condition, both at the immediately post‐exercise and delayed timepoints, than in either the moderate or all‐out‐HIIT conditions (all *p* < 0.05, Table 4).

Levels of BDNF in plasma also differed significantly between the three conditions, *χ* ^2^ (8) = 38.67, *p* < 0.001, conditional *R* ^2^ = 0.67 [0.46, 0.81]. Post‐hoc Tukey's tests of multiple comparisons demonstrated that levels of BDNF in plasma significantly increased from pre and post samples in all three conditions: moderate: 1610 [1100, 2120], *t*(143) = −3.17, *p* = 0.00531; *d* = −1.03 [−1.71, −0.36]; clinical‐HIIT: 1730 [767, 2690], *t*(143) = −3.23, *p* = 0.00435; *d* = −1.02 [−1.67, −0.37]; and all‐out‐HIIT: 1310 [586, 2040], *t*(143) = −2.46, *p* = 0.0399; *d* = −0.78 [−1.42, −0.13] (pg/mL); and significantly decreased from post to delayed samples in the moderate, *t*(143) = 3.57, *p = 0*.00141; *d* = 1.23 [0.52, 1.94], and clinical‐HIIT, *t*(143) = 3.71, *p* < 0.001; *d* = 1.19 [0.53, 1.85], conditions (Table 3). All effect sizes were moderate–large, *d* > 0.6. No other comparisons showed significant differences (Table 3; Table S1; Figure 3b; Table 4).

BDNF levels also differed significantly between conditions in platelet‐poor‐plasma, *χ* ^2^ (8) = 41.94, *p* < 0.001, conditional *R* ^2^ = 0.61 [0.40, 0.78]. Tukey's tests of multiple comparisons showed a significant increase in levels of BDNF in platelet‐poor‐plasma from pre to post samples in all conditions: moderate: 1460 [697, 2220], *t*(141) = −2.83, *p* = 0.0148; *d* = −0.94 [−1.61, −0.26]; clinical‐HIIT: 1690 [608, 2770], *t*(141) = −3.81, *p* < 0.001; *d* = −1.21 [−1.86, −0.56]; and all‐out‐HIIT: 1510 [628, 2390], *t*(141) = −4.56, *p* = 0.00242; *d* = −1.08 [−1.72, −0.43] (pg/mL). The levels of BDNF in platelet‐poor‐plasma also significantly decreased from post to delayed sample after clinical‐HIIT, *t*(141) = 4.42, *p* < 0.001; *d* = 1.42 [0.75, 2.09] (Table 3; Table S1; Figure 3c). All effect sizes were large, *d* > 0.8. No other comparisons showed significant differences (Table 3; Table S1; Figure 3b; Table 4).

There was no significant difference in the amount of BDNF per platelet between the three conditions, *χ* ^2^ (8) = 4.71, *p* = 0.788 (Figure 3d; Table 3; Table S1).

### 3.5 sAPPα

**FIGURE 4:**
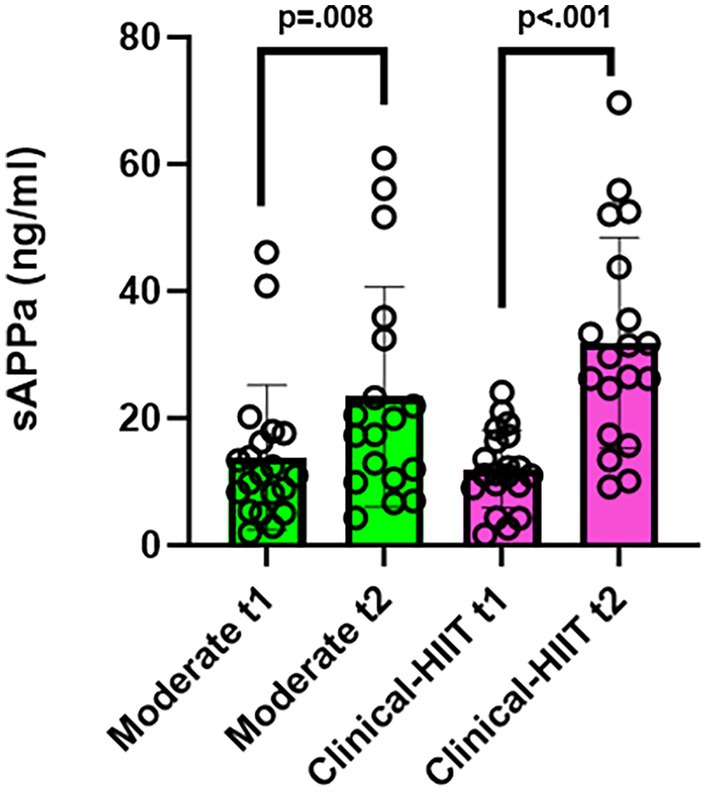
Amount of sAPPα (ng/mL). sAPPα in serum (ng/mL) for the moderate and clinical‐HIIT sessions. Note pre samples are denoted by t1; post by t2. Significant *p* values only shown, from Tukey's post‐hoc tests. Height of bars represents mean BDNF (pg/mL) and error bars represent standard deviation. All *N* = 20, except for moderate t2 (*N* = 18) and clinical‐HIIT t2 (*N* = 19), due to difficulties finding a suitable vein and one sample containing below detectable levels of protein.

The mixed effects model showed that there was a significant difference between levels of sAPPα between conditions, *χ* ^2^ (3) = 30.56, *p* < 0.001, conditional *R* ^2^ = 0.48 [0.28, 0.72]. The mean increase from baseline to post‐exercise in the moderate condition was 11.03 [5.24, 16.83]; and in the clinical‐HIIT condition was 19.47 [13.08, 25.87] ng/mL. Post‐hoc Tukey's tests of multiple comparisons found significant increases in levels of sAPPα from pre to post samples in both conditions: moderate, *t* (55) = −2.75, *p* = 0.00809, *d* = 0.90 [−1.57, −0.23]; and clinical‐HIIT, *t* (54) = −5.40, *p* < 0.001, *d* = −1.74 [−2.44, −1.03]. (Figure 4). Levels of sAPPα were significantly higher post‐exercise in the clinical‐HIIT condition than in the moderate condition, estimated Mean Difference = −7.58 [−14.90, −0.25], *p* = 0.0425, *d* = 0.67 [−0.00, 1.35].

## 4 DISCUSSION

This study aimed to investigate the impact of exercise intensity on neurotrophic factors across blood fractions in healthy young adults. BDNF increased in plasma and platelet‐poor plasma after exercise at moderate and high intensities (both clinical‐HIIT and all‐out‐HIIT sessions). In contrast, BDNF concentration in platelets was not altered by exercise, and in serum, BDNF increased after clinical‐HIIT and moderate intensity exercise alone. sAPPα increased after both moderate and clinical‐HIIT sessions.

In contrast to previous research, this study did not demonstrate that changes in BDNF were related to lactate, which might suggest that BDNF levels can increase independently of lactate production in the periphery and may be triggered by other mechanisms such as increased neuronal activity and cerebral blood flow (Smith et al., 1985; Walsh & Tschakovsky, 2018; more detail below). However, existing data have shown that infusion of lactate can produce an increase in BDNF (van der Steeg & Takken, 2021), which, when taken together with the findings presented herein, may indicate that BDNF is affected by other factors downstream of lactate production.

BDNF in serum increased immediately after the clinical‐HIIT and moderate intensity sessions, and was significantly higher in the clinical‐HIIT condition than in the moderate or all‐out‐HIIT condition post‐exercise. This finding agrees with previous research showing that BDNF levels in serum rise after acute aerobic exercise at moderate and high intensities (e.g., (Dinoff et al., 2018; Weaver et al., 2021)).

Serum accounts for BDNF that is both bound to platelets and unbound (Gejl et al., 2019), so should represent total levels of BDNF. The increase in levels of BDNF in serum may come from several sources. During exercise, there can be increased release of unbound BDNF from neurones and vascular endothelial cells (Walsh & Tschakovsky, 2018), which may cause an increase in peripheral levels of BDNF, as unbound BDNF is reported to cross the BBB (Pan et al., 1998). This is an adaptive response to exercise to promote the regulation of energy, tissue building, and modulation of muscle tone through remodeling (Ismail et al., 2020). BDNF can also be released from muscle in response to exercise, where it aids the regulation of fat metabolism (Cefis et al., 2023). However, levels of blood lactate, related to muscle metabolism (Schiffer et al., 2011), were unrelated to BDNF levels in serum, suggesting that muscle was not a primary source of BDNF release.

In plasma and platelet‐poor plasma, BDNF increased after exercise in all three conditions. Although BDNF levels in plasma and platelet‐poor plasma after exercise have not been thoroughly investigated, this finding is not concordant with previous research, which demonstrated that levels of BDNF in plasma were affected by exercise intensity (Reycraft et al., 2020; Weaver et al., 2021). Differences in methods of calculating exercise intensity and mode of exercise between these studies may explain these divergences. For example, (Weaver et al., 2021) calculated exercise intensity based on V̇O_2_ max and participants completed exercise on a treadmill, whereas the current study used maximum power output and cycling exercise.

Although platelets in plasma were not activated by the contents of the vacutainer tube, they may have been activated during exercise or inadvertently during sample withdrawal or preparation, thereby affecting BDNF concentration. Therefore, platelet‐poor plasma should provide the truest reflection of levels of unbound BDNF due to the removal of platelets during preparation. The increase seen in unbound BDNF after exercise is likely to be the result of increased release from neurones and vascular endothelial cells (Ismail et al., 2020), as discussed previously. Furthermore, high shear stress (the force exerted on blood vessel walls) has been associated with the release of 32% of total bound platelet BDNF, which may have also contributed to the reported increase in BDNF levels seen (Matthews et al., 2009). As previously noted, unbound BDNF in the periphery is thought to be able to cross the BBB (Pan et al., 1998) and thus increases in this form of BDNF after exercise may contribute to neuronal growth and improvements in cognition (Ishii & Nishida, 2013). The separation of plasma and platelet‐poor plasma here demonstrates that increases in unbound BDNF primarily contributed to increased BDNF levels in the periphery.

This study did not show an effect of exercise on levels of BDNF in platelets. Although previous work has demonstrated that levels of BDNF in platelets increased after exercise at V̇O_2_ max (Cho et al., 2012), little research has been conducted on how BDNF in platelets is affected by exercise at different intensities. The present findings suggest that platelet BDNF content is not significantly altered during exercise even under high intensity conditions in healthy young adults. This further suggests that the increases in BDNF seen in serum, plasma, and platelet‐poor plasma are likely to be the result of increased levels of unbound BDNF, rather than a transition from bound to unbound BDNF by increased platelet activity.

BDNF response to exercise was relatively transient, with BDNF levels showing no significant difference from baseline 30 min after the exercise bout was completed; in concordance with related research in this area (Walsh & Tschakovsky, 2018). Additionally, it is unclear at what timepoint during the exercise session that BDNF levels began to rise. This study was limited to acute bouts of exercise; however, there is some evidence to suggest that regular exercise is associated with increases in resting BDNF levels at baseline, and a more dramatic rise in BDNF levels after a single session of exercise (Szuhany et al., 2015). There is also some evidence to suggest that even transient increases in BDNF post‐exercise are associated with better performance on memory tasks, but findings are mixed (Piepmeier & Etnier, 2015).

These findings may be significant in relation to changes in BDNF levels throughout the day; though findings are conflicting on whether BDNF levels exhibit a circadian rhythm. (Ehrhardt et al., 2024) demonstrate that BDNF levels in plasma do not exhibit fluctuations as part of a circadian rhythm; though they suggest that BDNF levels in serum decrease significantly during the night. In contrast, (Cain et al., 2017) found that BDNF levels in plasma did exhibit circadian rhythms in some people; but further research is required to understand how exercise bouts affect levels of BDNF over a 24‐h period.

BDNF responses are not typically disaggregated by sex, and there is limited data on the impact of sex as a moderator on BDNF levels, with many studies in this area conducted in men alone (Dinoff et al., 2018; Szuhany et al., 2015). Therefore, recruitment in this study was open to males and females in order to further understand the sex‐specific activity of BDNF in each of the four blood fractions (Fujimura et al., 2002).

The response of BDNF to exercise was not shown to be affected by sex in this study in any of the blood fractions; providing evidence to suggest that males and females can both benefit from exercise‐induced BDNF increases. This contrasts with previous work which did find a sex difference in BDNF response post exercise. (Szuhany et al., 2015) demonstrated that BDNF levels post‐exercise increased less in women than in men irrespective of the blood fraction measured; whereas (Weaver et al., 2021) showed that BDNF levels in plasma after moderate and high intensity exercise were elevated more in females than in males. However, the analysis of sex differences is regarded as exploratory due to the limited number of females and consequent lack of power to detect any clinically important sex difference.

It is important to note that some populations with a higher risk of AD (e.g., those living with obesity or metabolic syndrome) have elevated resting levels of BDNF (Bacopoulou et al., 2023; Motamedi et al., 2017). This may be the result of impaired insulin resistance and glucose metabolism affecting BDNF expression (Bacopoulou et al., 2023). Repeated bouts of exercise in these groups can lower levels of serum BDNF (e.g., (Babaei & Azali Alamdari, 2013; Damirchi et al., 2014; Glud et al., 2019)). Therefore, while temporarily increasing BDNF levels after exercise or raising resting levels through regular exercise may benefit certain populations, it is important to consider an individual's needs regarding BDNF.

Taken together, the findings, particularly for the unbound portion of BDNF, are quite consistent across blood fractions. Serum reflects changes in BDNF response irrespective of platelet activation during exercise; whereas plasma, and to some extent platelet‐poor plasma, are likely to contain some BDNF that has been activated from platelets during exercise or sample preparation. Therefore, future research should be designed with an awareness of the effect of platelet response to exercise. Serum may be more robust to the process of sample preparation and able to detect differences in BDNF levels between sessions but could be less sensitive to detect subtle changes in BDNF within a session. If the research focus is on the mechanism of BDNF production, then it is important to consider all blood fractions; whereas if the focus is on BDNF change alone, serum may be a more cost‐effective measure.

Acute bouts of exercise raise levels of BDNF in healthy and clinical populations, including individuals with AD (e.g., Dinoff et al., 2018; Knaepen et al., 2010). Additionally, chronic exercise programmes in healthy individuals are associated with increased levels of BDNF at baseline (Szuhany et al., 2015). However, the effect of chronic exercise programmes on BDNF levels in clinical populations, particularly people with AD, is less well established. Further research is required to establish the impact of long‐term training programmes on neurotrophic factors in clinical populations.

sAPPα increased immediately after the moderate and clinical‐HIIT sessions and was higher post‐exercise in the clinical‐HIIT condition than in the moderate condition. To the best of our knowledge, this is the first study to examine the association between aerobic exercise in humans and sAPPα response. This extends findings from animal studies (e.g., (Koo et al., 2017; MacPherson et al., 2015)) which demonstrated that exercise increased levels of α‐secretase, contributing to increased sAPPα production in the brain. The finding here may show that increased processing of APP in the non‐amyloidogenic pathway occurs immediately after exercise, due to the increased levels of sAPPα found. There may be an effect of exercise intensity on sAPPα levels, but further research is required to investigate this.

The mechanism that drives increased sAPPα production after exercise remains unclear (Chan & Ye, 2017), as research in this area is still developing. However, previous research has demonstrated that increases in BDNF lead to increases in sAPPα in human cell models (Marko & MacPherson, 2022); increased activation of α‐secretase enzymes (Marko & MacPherson, 2022; Nigam et al., 2017), suggesting that the two are linked. Other exerkines, including the protease cathepsin B, inflammatory marker interleukin (IL) – 6, and lactate, have previously been associated with increased sAPPα production via inhibition of β‐secretase enzymes, enabling α‐secretases to metabolize more APP, in animal and cell studies (Piepmeier & Etnier, 2015), providing further mechanisms through which sAPPα production may be increased post‐exercise. The stimulation of BDNF production via exercise may offer a novel therapeutic method to shift APP processing towards the non‐amyloidogenic pathway, although further investigation is required to clarify the role of BDNF and other exerkines in promoting sAPPα production.

This study has some important limitations. As the participant group in this study was young, healthy, and physically active, it is difficult to assess whether the findings from this research can be replicated in different cohorts, including sedentary and older adult populations, who are likely to have a higher risk of AD. Therefore, further research is required to assess whether the findings can be reproduced in these populations. Additionally, further research is required to understand how exercise intensity affects BDNF release across the menstrual cycle. Female participants taking hormonal contraception were recruited into this study in order to limit the effect of hormonal variation between male and female participants and create a more homogenous participant group.

Additionally, participants exercised at moderate‐ and high‐intensities alone in this study. Therefore, the response of BDNF to low‐intensity exercise could not be examined; however, existing research suggests that low‐intensity exercise does not stimulate a BDNF response (e.g., (Schmidt‐Kassow et al., 2012)). Finally, the form of BDNF (pro or mature) was not measured here. Recent evidence suggests that the balance between pro and mature BDNF may become disrupted in aging (Cade et al., 2023), and proBDNF, but not mature BDNF expression, is increased in muscles after exercise (Edman et al., 2024). Further research is required to understand the effect the type of BDNF produced has on neuroplasticity, particularly in the context of aging and exercise.

In conclusion, this study demonstrated that exercise at moderate and high intensities can produce positive changes in neurotrophic factors, specifically BDNF and sAPPα, which are relevant to AD. Serum offers the most reliable and representative blood fraction for measurement of BDNF; however, serum alone may not be sufficient to fully understand the mechanism of response of BDNF to exercise. Further research is required to explore whether sAPPα production can be stimulated after exercise in adults at risk of AD.

## AUTHOR CONTRIBUTIONS

All experiments were performed in the School of Sport, Exercise, and Rehabilitation Sciences at the University of Birmingham. FS: Conceptualization; Methodology; Validation; Formal analysis; Investigation; Writing – Original Draft; Writing – Review and Editing; Visualization; Project administration; RE: Conceptualization; Methodology; Investigation; Writing – Review and Editing; Visualization; Project administration; Supervision LB: Conceptualization; Writing – Review and Editing; Supervision; JB: Conceptualization; Formal analysis; Writing – Review and Editing; Supervision; SL: Conceptualization; Writing – Review and Editing; Supervision; SA: Conceptualization; Methodology; Writing – Review and Editing; Supervision; Project administration.

## FUNDING INFORMATION

FS received funding provided by the MRC Trials Methodology Research Partnership (TMRP) Doctoral Training Partnership (DTP). Grant Number: MR/W006049/1.

## CONFLICT OF INTEREST STATEMENT

The authors declare that there are no conflicts of interest.

## DISCLAIMERS

The authors confirm that there are no disclaimers to declare.

## Supporting information


Table S1.

## Data Availability

Source data for this study are openly available at https://doi.org/10.25500/edata.bham.00001239.
