# Supplementary material for: Brain derived neurotrophic factor and secreted amyloid precursor protein‐α response to moderate and high intensity exercise
Source: Physiol Rep. 2025 May 22;13(10):e70366. doi: 10.14814/phy2.70366 (PMC12099066; doi:10.14814/phy2.70366)
Supplement: Supplementary file 1 — Table S1. [file PHY2-13-e70366-s001.docx]

Supplementary Information

Supplementary Table 1.Results of Tukey’s tests of multiple comparisons for blood lactate, and BDNF in serum, plasma, and platelet-poor-plasma, within sessions.

| **Lactate** | | | | | | |
| --- | --- | --- | --- | --- | --- | --- |
| **Overall Model**  **[95% CI]** | **Fixed Effects** | **Post Hoc Tests**  **[95% CI]** | | | | |
| Χ^2^ (8) = 296.96, *p*<.001  Conditional R^2^ = 0.84 [0.80, 0.89] | Condition: F(2, 143) = 0.08, *p*=.928  Time: F(2, 143)= 175.84, *p*<.001  Condition * Time: F(4, 143) = 29.66, *p*<.001 |  | Moderate | Clinical-HIIT | | All-out-HIIT |
|  |  | Pre vs Post | **t(143) = -4.71, *p<*.001; d = -1.53 [ -2.19, -0.86]** | **t(143) = -17.66, *p*<.001; d = -5.73 [-6.63, -4.83]** | | **t(143) = -18.10, *p*<.001; d = -5.72 [-6.61, -4.83]** |
|  |  | Post vs Delayed | **t(144) = 3.82, *p*=.00100; d = 1.26 [0.59, 1.92]** | **t(143) = 12.84, *p*<.001; d = 4.17 [3.38, 4.96]** | | **t(144) = 13.08, *p*<.001, d = 4.26 [3.46, 5.06]** |
|  |  | Pre vs Delayed | t(144) = -0.82, *p*=.691; d = -0.27 [-0.92, 0.38] | **t(143) = -4.81, *p*<.001; d = -1.56 [-2.23, -0.90]** | | **t(144) = -4.49, *p*<.001; d = -1.46 [-2.13, -0.80]** |
| **Serum BDNF** | | | | | | |
| Χ^2^ (8) = 49.71, *p*<.001  Conditional R^2^ = 0.49 [0.25, 0.63] | Condition: F(2, 143) = 3.27, *p*=.0409  Time: F(2, 143)= 2.47, *p*=.0682  Condition * Time: F(4, 143) = 0.24, *p*=.913 |  | Moderate | Clinical-HIIT | All-out-HIIT | |
|  |  | Pre vs Post | **t(143) = -2.72, *p*=.0200; d =0.89 [ -1.54, -0.23]** | **t(143) = -3.31, *p*=.00333; d = -1.05 [-1.69, -0.41]** | t(143) = -2.13, *p*=.0882; d = -0.67 [-1.31, -0.04] | |
|  |  | Post vs Delayed | t(143) = 2.21, *p*=.0727; d = 0.76 [0.07, 1.45] | t(143) = 2.01, *p*=.112; d = 0.65 [0.00, 1.29] | t(143) = 1.89, *p*=.147, d = 0.62 [-0.04, 1.27] | |
|  |  | Pre vs Delayed | t(144) = -0.37, *p*=.928; d = -0.12 [-0.80, 0.55] | t(143) = -1.25, *p*=.425; d = -0.40 [-1.04, 0.24] | t(143) = -0.18, *p*=.983; d = -0.06 [-0.71, 0.59] | |
| **Plasma BDNF** | | | | | | |
| Χ^2^ (8) = 38.67, *p*<.001  Conditional R^2^ = 0.67 [0.46, 0.81] | Condition: F(2, 143) = 0.50, *p*=.608  Time: F(2, 143)= 3.51, *p*=.0326  Condition * Time: F(4, 143) = 0.45, *p*=.770 |  | Moderate | Clinical-HIIT | All-out-HIIT | |
|  |  | Pre vs Post | **t(143) = -3.17, *p*=.00531; d =-1.03 [ -1.71, -0.36]** | **t(143) = -3.23, *p*=.00435; d = -1.02 [-1.67, -0.37]** | **t(143) = -2.46, *p*=.0399; d = -0.78 [-1.42, -0.13]** | |
|  |  | Post vs Delayed | **t(143) = 3.57, *p=*.00141; d = 1.23 [0.52, 1.94]** | **t(143) = 3.71, *p<*.001; d = 1.19 [0.53, 1.85]** | t(143) = 2.05, *p*=.103, d = 0.67 [-0.01, 1.33] | |
|  |  | Pre vs Delayed | t(144) = -0.59, *p*=.828; d = 0.20 [-0.49, 0.88] | t(143) = -0.42, *p*=.861; d = 0.17 [-0.48, 0.82] | t(143) = -0.33, *p*=.942; d = -0.11 [-0.77, 0.55] | |
| **Platelet-Poor-Plasma BDNF** | | | | | | |
| Χ^2^ (8) = 41.94, *p*<.001  Conditional R^2^ = 0.61 [0.40, 0.78] | Condition: F(2, 141) = 0.57, *p*=.567  Time: F(2, 141)= 5.87, *p*=.00356  Condition * Time: F(4, 141) = 1.41, *p*=.233 |  | Moderate | Clinical-HIIT | All-out-HIIT | |
|  |  | Pre vs Post | **t(141) = -2.83, *p*=.0148; d =-0.94 [ -1.61, -0.26]** | **t(141) = -3.81, *p<*.001; d = -1.21 [-1.86, -0.56]** | **t(141) = -4.56, *p*=.00242**; **d = -1.08 [-1.72, -0.43]** | |
|  |  | Post vs Delayed | t(141) = 2.00, *p*=.116; d = 0.70 [-0.01, 1.41] | **t(141) = 4.42, *p*<.001; d = 1.42 [0.75, 2.09]** | t(141) = 1.38, *p*=.355, d = 0.45 [-0.21, 1.11] | |
|  |  | Pre vs Delayed | t(142) = -0.66, *p*=.785; d = - 0.23 [-0.93, 0.47] | t(141) = -0.67, *p*=.783; d = 0.21 [-0.43, 0.86] | t(141) = -1.93, *p*=.134; d = -0.63 [-1.29, 0.03] | |
| **BDNF Per Platelet** | | | | | | |
| Χ^2^ (8) =4.71, p=.788  Conditional R^2^ = 0.14 [0.06, 0.37] | Condition: F(2, 130) = 0.05, *p*=.948  Time: F(2, 129)= 1.05, *p*=.354  Condition * Time: F(4, 130) = 0.49, *p*=.741 |  | Moderate | Clinical-HIIT | All-out-HIIT | |
|  |  | Pre vs Post | t(130) = -0.14, *p*=.989; d = -0.05 [ -0.72, 0.62] | t(129) = 0.45, *p*=.897; d = 0.15 [-0.52, 0.82] | t(129) = 0.17, *p*=.985; d = 0.05 [-0.60, 0.70] | |
|  |  | Post vs Delayed | t(131) = 0.27, *p*=.960; d = 0.70 [-0.01, 1.41] | t(130) = -0.03, *p*=.990; d = -0.01 [-0.69, 0.67] | t(129) = -1.18, *p*=.469, d = -0.44 [-1.11, 0.22] | |
|  |  | Pre vs Delayed | t(131) = 0.14, *p*=.990; d = 0.05 [-0.67, 0.77] | t(131) = 0.41, *p*=.913; d = 0.14 [-0.53, 0.81] | t(129) = -1.32, *p*=.385; d = -0.39 [-1.04, 0.27] | |
